# Deep brain stimulation in Lesch–Nyhan disease: outcomes from the patient’s perspective

**DOI:** 10.1111/dmcn.14852

**Published:** 2021-03-10

**Authors:** JASPER E VISSER, ADAM C COTTON, DAVID J SCHRETLEN, JOCELYNE BLOCH, KRISTINA TEDROFF, GASTÓN SCHECHTMANN, DIANA RADU DJURFELDT, VICTORIA GONZALEZ, LAURA CIF, HYDER A JINNAH

**Affiliations:** 1 Department of Neurology, Donders Institute for Brain, Cognition and Behavior, Radboud University Medical Center, Nijmegen; 2 Department of Neurology, Amphia Hospital, Breda, the Netherlands; 3 Departments of Neurology and Human Genetics, Emory University School of Medicine, Atlanta; 4 Department of Psychiatry and Behavioral Sciences, Johns Hopkins University School of Medicine, Baltimore, USA; 5 Department of Neurosurgery, Lausanne University Hospital and University of Lausanne, Lausanne, Switzerland; 6 Neuropediatric Unit, Department of Women’s and Children’s Health, Karolinska Institutet and Karolinska University Hospital, Stockholm; 7 Department of Neurosurgery, Karolinska Institutet and University Hospital, Stockholm; 8 Department of Clinical Neuroscience, Karolinska Institutet, Stockholm, Sweden; 9 Department of Neurology, CHU Montpellier and INSERM U661, Montpellier; 10 Department of Neurosurgery, CHU Montpellier, Montpellier, France

## Abstract

**AIM:**

To provide insight into outcome and long-term safety and efficacy of deep brain stimulation (DBS), from the perspective of individuals with Lesch–Nyhan disease (LND) and their families.

**METHOD:**

We used patient-centered outcome measures to assess long-term outcomes of DBS for 14 individuals (mean [SD] age 10y 10mo [5y 6mo], range 5–23y, all males) with LND, after an average duration of 5y 6mo (range 11mo–10y 5mo) after surgery. We compared these results with a comprehensive review of previously published cases.

**RESULTS:**

Patients and their families reported that DBS of the globus pallidus can be effective both for motor and behavioral disturbances in LND. However, outcome measures were often not significantly changed owing to substantial variability among individuals, and were overall less positive than in previous reports based on clinician assessments. In addition, there was an unexpectedly high rate of adverse events, tempering overall enthusiasm for the procedure.

**INTERPRETATION:**

Although DBS might be an effective treatment for LND, more research is needed to understand the reasons for response variability and the unusually high rates of adverse events before DBS can be recommended for these patients.

Lesch–Nyhan disease (LND) is caused by loss of the purine salvage enzyme hypoxanthine-guanine phosphoribosyl-transferase, leading to hyperuricemia and a distinctive neurobehavioral phenotype.^1,2^ The movement disorder is dominated by dystonia, although chorea and spasticity are sometimes also present.^3^ Intellectual disability involves mainly executive tasks and attention.^4^ Behavioral abnormalities include oppositional and severe self-injurious behavior.^5,6^ Patients with partial enzyme deficiency (LND variants) do not exhibit self-injurious behavior, although the movement disorder and intellectual disability may occur with variable severity.^7^

Biochemical,^8,9^ histopathological,^10^ functional imaging,^11,12^ and experimental^13–15^ sources of evidence have indicated that the neurobehavioral abnormalities result from dysfunction of the basal ganglia, and especially the dopaminergic pathways.^16^ Existing treatments are only partly successful. Of note, levodopa does not provide a useful treatment, despite the profound dopamine deficiency in LND.^17^ Numerous other medications have been tried, with minimal success.^1^ Self-injury can often only be controlled by using protective straps to hold down the limbs. However, in recent years, deep brain stimulation (DBS) of the globus pallidus has been reported to reduce the severity of dystonia and reduce self-injurious behavior in LND, in several small studies.^18–26^

The current study provides a comprehensive review of all 12 published cases, followed by an assessment of outcomes from the perspective of 14 participants and their families.

## METHOD

For the literature review, the PubMed database was queried for articles that included the keywords ‘Lesch–Nyhan’ and ‘deep brain stimulation’ (last checked on 1st October 2020). From the results and reference lists therein, nine publications were identified, describing 12 unique cases. All were included here. Several large reviews have been published where LND cases were summarized. These cases were not included here because insufficient clinical data were provided, and some of the cases reviewed were already published as case reports.

For the assessment of outcomes, 14 participants were identified through direct patient contact, patient support networks, or the Lesch–Nyhan Disease International Study Group. These participants came from France, the Netherlands, Sweden, Switzerland, or the USA. All received their procedures at centers with extensive experience with DBS. Data were assembled after review by the Medical Research Ethics Committee (Committee on Research Involving Human Subjects [CMO], Arnhem-Nijmegen, the Netherlands). Owing to the nature of the study, informed consent was not required. Early findings were previously reported for two of these cases, as included in the literature review.^20,23,24^

Primary caregivers were asked to discuss the procedure and its outcomes with the participant and other family members. Six individuals were not continuing with DBS at the time of reporting. The caregivers then completed a standardized questionnaire, which was adapted from a previous study of LND (Appendix S1, online supporting information).^17^ This questionnaire included 20 items using 9-point Likert-type scales.^27^ The first 18 questions assessed changes after DBS in six neurobehavioral domains (three questions per domain), including abnormal movements, self-injurious behavior, oppositional behavior, apathy, agitation, and depression. The answers were averaged to give a single result for each domain for each participant. Additional questions assessed overall opinions: the overall effect of DBS, whether they would consider DBS again in another patient with LND, and the participant’s own opinion about the DBS.

Data about adverse events were also methodically collected and tabulated. Finally, caregivers were asked to take into account all benefits and adverse events and give an overall assessment of the procedure, such as whether they would repeat it if needed, or recommend it to other individuals with LND.

### Statistical analysis

Scores on the neurobehavioral domains and additional questions were analysed for their median (to assess the magnitude of changes) and interquartile range (IQR; as a measure of variability among respondents) using SPSS version 27.0 (IBM Corporation, Armonk, NY, USA) and Box-PlotR.^28^

## RESULTS

### Literature search

Results from 12 unique LND cases previously reported are summarized in Table 1. The average reported age was 14 years 1 month (range 5y 5mo–28y). All individuals received bilateral stimulation of the globus pallidus. Improvements in dystonia were reported for all patients. Quantitative assessments of improvements in dystonia using the Burke–Fahn–Marsden Dystonia Rating Scale^29^ scores were provided for eight cases, where there was a mean (SD) improvement of 19.7% (20.5%).

All reports also described improvements in self-injurious behavior. This behavior, which is normally a daily occurrence in LND,^4,5^ was reported to disappear in six cases, and became ‘rare’ in another. The remaining cases showed 50% to 80% reductions in the frequency and severity of difficult behaviors (including self-injurious behavior) using the Behavior Problems Inventory rating scale.^30^ Reported complications were addressed in only three papers, limited to hardware failures and infections in six participants.

### Patient-centered outcomes

Outcomes for DBS surgery were collected from primary caretakers for 14 participants (Table 2). Thirteen participants were diagnosed with classic LND, on the basis of residual hypoxanthine-guanine phosphoribosyltransferase enzyme activity, *HPRT1* gene analysis, and/or the full clinical phenotype with hyperuricemia, dystonia, and self-injurious behavior. One participant had dystonia with intellectual disability but did not express self-injurious behavior, and therefore met the criteria for being a variant without self-injury.^7^ Average age at the time of surgery was 10 years 10 months (range 5–23y). All patients underwent bilateral DBS of the globus pallidus. Seven had two electrodes implanted on each side (a total of four electrodes per case), to treat motor and behavioral aspects of the phenotype separately. The questionnaires were received on average 5 years 6 months after surgery (range 11mo–10y 5mo).

**Figure 1:**
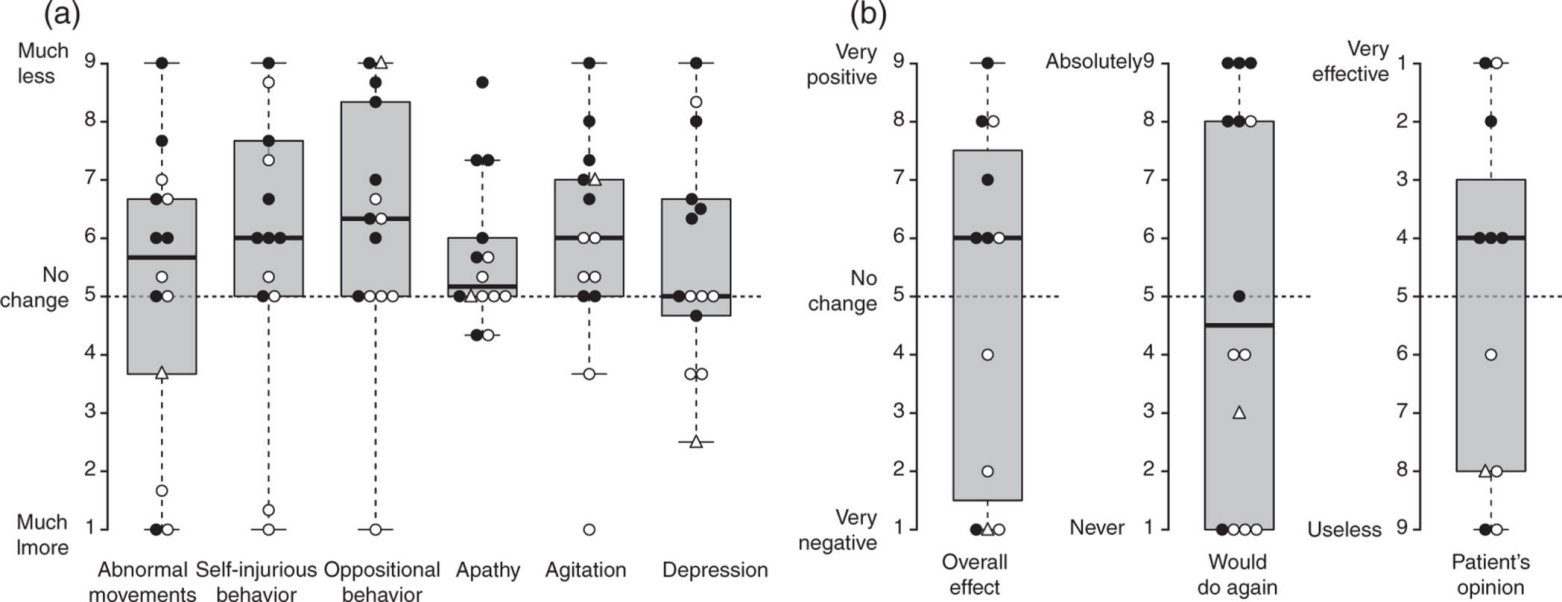
Summary of caregiver assessments (a) about the effects of deep brain stimulation (DBS) in Lesch–Nyhan disease (LND) across six neurobehavioral domains and (b) answers to three questions assessing overall opinions about this treatment. In (a), each symbol shows an individual patient’s mean score for three questionnaire items that assess the respective domain. In (b), each symbol shows the score for the respective patient. Center lines show the medians; box limits indicate the 25th and 75th centiles; whiskers extend 1.5 times the interquartile range from the 25th and 75th centiles. Symbols: circle, LND; triangle, LND variant; closed symbols, two electrodes each side; open symbols, one electrode each side.

For the entire group of 14 participants, median rating scale scores were above 5 (suggesting improvements across most survey domains) (Fig. 1a), including abnormal movements (median 5.7, IQR 3.0), self-injurious behavior (median 6.0, IQR 2.7), oppositional behavior (median 6.3, IQR 3.3), apathy (median 5.2, IQR 1.0), and agitation (median 6.0, IQR 2.0). The median for depression indicated no change (median 5.0, IQR 2.0). For all of these domains, the median was consistently either 5 or very close to 5, suggesting that a clinically important change in any of these domains was lacking. Perhaps more importantly, the wide IQRs for most of the measures resulted from considerable variation in individual responses.

All patients but one experienced at least one adverse event (Table 2), either during the initial perioperative period or during follow-up. Six patients developed infections involving the equipment. Only one of these infections was attributed to persistent self-injurious behavior directed to the head. Five patients experienced hardware-related complications, such as broken wires. All but two patients required at least one additional surgical procedure.

Taking into account these benefits and adverse events, the overall impressions from caretakers were mixed (Fig. 1b). About half of the caregivers were positive about the overall effect of DBS in LND to a variable degree (median score 6.0, IQR 6.0), and only a similar proportion endorsed repeating the procedure (median 4.5, IQR 7.0). Also, about half of the patients were positive about the effects of DBS (median 6.0, IQR 5.0). Again, very wide IQRs indicated the absence of any consistent pattern, owing to extreme variability.

## DISCUSSION

This study reviews reported effects of DBS in LND and compares them with patient-centered outcome measures to: (1) capture direct evidence of the perceived treatment benefit and (2) evaluate the utility of DBS for potential future patients.

The literature review including eight patients paints a very positive picture of the application of DBS in LND. Often, marked benefits in self-injurious behavior and at least partial benefits in the motor disorder were noted, and significant adverse events were infrequently reported. Our assessment of the outcomes of 14 patients from the viewpoint of the patients and their families provides a different perspective. In summary, perceived benefits varied considerably among responders and adverse events were common. Although many families gave an overall favorable assessment of the benefits of DBS, only about half would repeat the procedure.

The reasons for the variable overall impressions of DBS in LND by families cannot be conclusively determined from this study. However, a first possible explanation for the varied outcomes involves technical aspects of surgical implantation and programming. For example, the target site varied and several cases received two electrodes per side (four electrodes in total) in an attempt to specifically address both motor and non-motor features of LND by stimulating motor and limbic regions of the globus pallidus simultaneously. A review of the data (Fig. 1a,b), however, indicated no apparent differences among participants who had four versus two electrodes per side. All cases were operated and programmed at experienced centers, so it seems unlikely that lack of experience explains the variability. Another possible explanation for the variable motor benefit is that the movement disorder in LND and its variants is variable and mixed.^3,7^ Dystonia is the dominant feature, but severity is variable among participants, and some cases also have spasticity and chorea. Although the magnitude of previously reported effects of DBS on the dystonia in LND seems to fall in the range that has been reported before for other dystonias,^31,32^ the other motor problems in LND that differ among participants may not respond to DBS of the internal globus pallidus and variably limit its effect. A third explanation for variable treatment effects may relate to the age of the participants at operation, which varied considerably. Presumably, treatment of the motor disorder at later ages and/or after longer disease duration may not be reversed as readily. Indeed, both younger age at time of surgery and shorter duration of symptoms were associated with better DBS outcomes,^31,33^ whereas longer duration of dystonia symptoms correlated negatively in previous studies.^34^

The reasons for the high frequencies of adverse events also remains uncertain. Even considering that DBS surgical site infections are more frequent in children compared with adults,^19,35^ the high rate of infections is of note, as LND is not associated with any known defect in the immune system. Also, the rate of hardware-related problems (50%) is substantially higher than previously reported in children (about 13–18%).^19,35^ Self-injurious behavior directed towards the equipment was reported in one patient only, and therefore does not appear to provide a good explanation for infections or equipment failures as has been postulated before.^26^ It is of note that, in this study, almost all patients underwent additional surgical interventions to treat complications or because of lack of effect. The serious consequence of hardware failures in patients with LND is clear, as the reoccurrence of severe dystonia^32^ and self-injurious behavior after sudden cessation of DBS may become medical emergencies.

To explain the discrepancy in the very positive outcomes reported in previous studies and the variable benefits with frequent adverse events in the current study, it is important to recognize that the literature on case reports is well-known for being biased towards favorable outcomes.^36^ Cases with lack of benefit or negative outcomes are less commonly reported, and negative follow-up reports are usually not published. In addition, the assessments of the investigators that publish the reports sometimes may not match the impressions of the patients or their families. Nevertheless, it has been shown that case reports have a significant influence on subsequent publications and possibly on clinical practice as well.^37^ Therefore, it is crucial that anecdotal observations are tested in controlled clinical trials to guide future clinical practice.

Several limitations of this study should be acknowledged. One is recall bias. Although a certain degree of recall bias cannot be excluded, it must be noted that in children where the DBS is still on, the questionnaire addresses the current effects of the treatment, thus recall is not relevant. Moreover, most of the adverse events are less likely to be affected by recall bias, because most required additional procedures that could be verified in the medical records. Another limitation is the small number of participants, operated at different centers using different techniques. Although this factor may have contributed to variability in the results, it also shows a more representative result about what happens in the broader community, rather than at a single site. Third, the patient-centered outcome measures were obtained by giving the patient or family a questionnaire to fill out, or by reading the questionnaire to the patient and family as an aid to understanding the questions. The examiner was allowed to answer questions, but was not allowed to influence the result. This strategy may have influenced the result, although reports of adverse events are unlikely to be omitted or exaggerated by the presence of an examiner. Finally, the questionnaire used was not formally validated. Because LND is extremely rare with a very distinctive neurobehavioral phenotype, there are no formally validated clinician rating scales or patient-centered rating scales available. However, we followed standard recommended procedures when designing the questionnaire.^27^

In summary, individuals with LND and their families report very variable outcomes in the influence of DBS on the behavior and motor disorder of LND. Some patients report good outcomes, whereas others do not. Adverse events requiring re-operation are common. Thus, although DBS might be an effective treatment for LND, more research is needed to fully understand the risk/benefit ratio for DBS in LND before DBS can be recommended for these patients. Ideally, these studies should be conducted by experienced teams prepared to deal with adverse events in this difficult population. In the meantime, the impressions of patients and families and the high risk of adverse events should be taken into consideration when counseling potential future patients and families about this treatment option.

## Supplementary Material

Appendix S1

## Figures and Tables

**Table 1:** Summary of the literature for DBS in Lesch–Nyhan disease

Study	Age at surgery (y)	Follow-up (y:mo)	Target(s) of DBS	Effect on dystonia	Effect on behavior	Complications
Air et al.^19^	5	1:0	Bilateral GPi	16% improvement on BFMDRS	Decrease on BPI of 80% (frequency) and 75% (severity)	Lead fracture
Deon et al.^21^	10	2:6	Bilateral GPi	Dystonia decreased, comfort and function improved within 3mo	Self-injurious behavior disappeared	None
Tambirajoo et al.^26^	11	1:10	Bilateral posterior GPi	1.6% improvement on BFMDRS movement scale, 0% on disability scale	Mild and temporary improvement	Migration of a lead infection
Pralong et al.^23^	12	NR	Bilateral anterior and posterior GPi	‘Significant decrease’ of dystonic movements	Disappearance of many self-injurious behaviors within 3mo	NR
Pralong et al.^23,24^	12	NR	Bilateral anterior and posterior GPi	‘Significant decrease’ of dystonic movements	Disappearance of many self-injurious behaviors within 3mo	NR
Tambirajoo et al.^26^	12	3:1	Bilateral anterior and posterior GPi	6.7% improvement on BFMDRS movement scale, 8.3% on disability scale	Improvement in frequency and severity of self-injurious behavior	Infection
Tambirajoo et al.^26^	13	8:1	Bilateral anterior and posterior GPi	3.8% improvement on BFMDRS movement scale, 6.9% on disability scale	Decrease on BPI of 68% (frequency) and 78% (severity) after 1y follow-up	Infection
Abel et al.^18^	15	0:6	Bilateral GPi	Moderate improvement over course of several weeks	Self-injurious behavior became rare	Lead fracture and dislocations after fall
Cif et al.^20^	16	2:4	Bilateral anterior and posterior GPi	40% improvement on BFMDRS, up to 28mo	Self-injurious behavior disappeared within several days	NR
Tambirajoo et al.^26^	16	12:0	Bilateral anterior and posterior GPi	1.3% improvement on BFMDRS movement scale, 4.0% on disability scale	Decrease on BPI of 53% (frequency) and 50% (severity) after 1y follow-up	Hardware issues
Taira et al.^25^	19	2:0	Bilateral GPi	Gradual improvement, 33% reduction of BFMDRS	Self-injurious behavior disappeared after 3mo	NR
Piedimonte et al.^22^	28	5:0	Bilateral GPi	55% decrease in BFMDRS	Self-injurious behavior disappeared within months	None

DBS, deep brain stimulation; GPi, internal globus pallidus; BFMDRS, Burke–Fahn–Marsden Dystonia Rating Scale; BPI, Behavior Problems Inventory; NR, not reported.

**Table 2:** Demographic data and adverse events reported after DBS in each of the 14 cases for whom long-term assessments were collected

Case	Clinical phenotype	Age at surgery (y)	Follow-up (y:mo)	Number of electrodes per side	Electrode location(s)	Problem(s)	Consequence	DBS still active at time of assessment
1	LND	7	7:9	2	GPi, ventral pallidum	Broken wire	Replaced	Yes
2	LND	5	3:2	1	GPi	Lead infection, broken wires	Electrode replaced	Yes (one side only)
3	LND	11	4:9	2	GPi	Faulty equipment	Replaced	Yes
4	LND	6	3:3	2	GPi	Scalp infection spread to equipment	Removed one lead	Yes (one side only)
5	LND	12	5:8	2	GPi, anterior and posterolateral	Scalp infection due to self-injurious behavior causing cerebral abscess	Removed	No
6	LND	5	7:8	1	GPi	Epilepsy, CSF leakage, local pain, infection, swallowing difficulties	Replaced, turned off	No
7	LND	13	5:3	1	GPi	Skin erosion, wires exposed, infection, surgical removal	Removal	No
8	LND	12	10:4	2	GPi, ventral pallidum	Delayed hardware failure, broken extension cable, infection	Replaced	Yes
9	LND	11	8:11	2	GPi, ventral pallidum	Not reported	Two stimulator replacements	Yes
10	LND	7	1:6	1	GPi	Seizure, edema around electrodes, stimulator pack improperly placed	Re-surgery to correct stimulator placement	Yes
11	LND	23	9:9	1	GPi	None reported	Discontinued	No^a^
12	LND	21	4:5	2	GPi	Uncontrolled movements, speech problems	Turned off	No
13	LND	11	0:11	1	GPi	Confusion, extreme movements, speech difficulties		Yes
14	LND variant	7	3:9	1	GPi	Abnormal sensations	Removed	No

a Owing to lack of effect. DBS, deep brain stimulation; LND, Lesch–Nyhan disease; GPi, globus pallidus internal segment; CSF, cerebrosp-inal fluid.

## What this paper adds

- Individuals with Lesch–Nyhan disease and their families report variable efficacy of deep brain stimulation.
- Long-term outcomes are associated with a high adverse event rate.

## ABBREVIATIONS

DBS: Deep brain stimulation
LND: Lesch–Nyhan disease
