# Supplementary material for: Deep brain stimulation in Lesch–Nyhan disease: outcomes from the patient’s perspective
Source: Dev Med Child Neurol. 2021 Mar 10;63(8):963–8. doi: 10.1111/dmcn.14852 (PMC8350791; doi:10.1111/dmcn.14852)
Supplement: Supplementary file 1 — Appendix S1: Retrospective evaluation of deep brain stimulation for Lesch–Nyhan disease. [file DMCN-63-963-s001.pdf]

Retrospective evaluation of deep brain stimulation for Lesch-Nyhan disease

Part 1. Procedure

Patient name : ______ **Date of birth :** ______

Date(s) of surgery (implantation) : ______

Type of stimulator : ______ ☐ Unknown

The electrodes were implanted : ○ Unilaterally (i.e. one side of the brain), namely : ○ Left ○ Right
○ Bilaterally (i.e. both left and right)

How many electrodes were implanted in *each side of the brain* : ○ 1 ○ 2

In which brain area were the electrodes implanted?*: ☐ Globus pallidus ☐ Ventral pallidum ☐ Subthalamic nucleus ☐ Thalamus ☐ Other : ______ ☐ Unknown

Where there any complications in the period of surgery : ○ No ○ Yes

If yes, what happened* : ☐ Visual disturbances ☐ Eye movement difficulties ☐ Infections ☐ Speech or language problems ☐ Swallowing problems ☐ Stroke ☐ Paresis (muscle weakness) ☐ Sensory problems ☐ Intracerebral bleeding ☐ Extreme, wild movements` ☐ Epilepsy ☐ Local pain ☐ Emotional lability ☐ Confusion ☐ Other: ______

Where there any changes in medication while on deep brain stimulation? ○ Yes ○ No

If yes, what was changed: ______

Have the electrodes ever been removed? ○ Yes ○ No

If so, the reason to remove them was: ______

If so, have they been put back, or are there plans to do so ? ○ Yes ○ No

Is the stimulator still on? ○ Yes ○ No

If not, the reason to turn off was: ______

Please answer the following questions using the scale shown to the right of each item. ***For each blank space below tick one number that best represents your opinion.***

			1	2	3	4	5	6	7	8	9	
1.	In retrospect, the procedure of DBS was …..	Trouble-some	○	○	○	○	○	○	○	○	○	Effort-less
2.	In retrospect, the effect of DBS was …..	Very negative	○	○	○	○	○	○	○	○	○	Very positive
3.	The powerpack or subcutaneous electrodes triggered new forms of self-injury aimed at the equipment.	Never	○	○	○	○	○	○	○	○	○	Ex-tremely
4.	I would ….. consider deep brain stimulation again in another child/patient with LND.	Never	○	○	○	○	○	○	○	○	○	Abso-lutely

Any other remarks regarding the procedure :

______

** More than one answer is possible*

Retrospective evaluation of deep brain stimulation for Lesch-Nyhan disease

Part 2. Effect

Please compare the patient's behavior with deep brain stimulation (turned on) to his usual behavior before he was operated. *For each blank space below tick one number that best represents the patient's behavior, attentiveness, muscle stiffness, and mood compared to the period before deep brain stimulation commenced.*

#	Behavior or characteristic being rated	Rating										
1.	The patient tried to injure himself ….. than before deep brain stimulation started	Much more	1 ○	2 ○	3 ○	4 ○	5 ○	6 ○	7 ○	8 ○	9 ○	Much less
2.	The patient tried to injure others ….. than before deep brain stimulation started	Much more	1 ○	2 ○	3 ○	4 ○	5 ○	6 ○	7 ○	8 ○	9 ○	Much less
3.	The patient is ….. interactive than before deep brain stimulation started	**Much less**	1 ○	2 ○	3 ○	4 ○	5 ○	6 ○	7 ○	8 ○	9 ○	**Much more**
4.	The patient showed ….. muscle stiffness than before deep brain stimulation started	Much more	1 ○	2 ○	3 ○	4 ○	5 ○	6 ○	7 ○	8 ○	9 ○	Much less
5.	The patient showed ….. agitation than before deep brain stimulation started	Much more	1 ○	2 ○	3 ○	4 ○	5 ○	6 ○	7 ○	8 ○	9 ○	Much less
6.	The patient showed ….. optimism than before deep brain stimulation started	**Much less**	1 ○	2 ○	3 ○	4 ○	5 ○	6 ○	7 ○	8 ○	9 ○	**Much more**
7.	The patient showed ….. self-abusive behavior than before deep brain stimulation started	Much more	1 ○	2 ○	3 ○	4 ○	5 ○	6 ○	7 ○	8 ○	9 ○	Much less
8.	The patient showed ….. abusive behavior toward others than before deep brain stimulation started	Much more	1 ○	2 ○	3 ○	4 ○	5 ○	6 ○	7 ○	8 ○	9 ○	Much less
9.	The patient showed ….. apathy than before deep brain stimulation started	Much more	1 ○	2 ○	3 ○	4 ○	5 ○	6 ○	7 ○	8 ○	9 ○	Much less
10.	The patient showed ….. jerky movements than before deep brain stimulation started	Many more	1 ○	2 ○	3 ○	4 ○	5 ○	6 ○	7 ○	8 ○	9 ○	Many fewer
11.	The patient showed ….. calm patience than before deep brain stimulation started	**Much less**	1 ○	2 ○	3 ○	4 ○	5 ○	6 ○	7 ○	8 ○	9 ○	**Much more**
12.	The patient showed ….. sadness than before deep brain stimulation started	Much more	1 ○	2 ○	3 ○	4 ○	5 ○	6 ○	7 ○	8 ○	9 ○	Much less
13.	The patient needed ….. frequent restraints to prevent self-injury than before deep brain stimulation started	Much more	1 ○	2 ○	3 ○	4 ○	5 ○	6 ○	7 ○	8 ○	9 ○	Much less
14.	The patient showed ….. oppositional, defiant, sneaky, or deceptive behavior than before deep brain stimulation started	Much more	1 ○	2 ○	3 ○	4 ○	5 ○	6 ○	7 ○	8 ○	9 ○	Much less
15.	The patient showed ….. indifference than before deep brain stimulation started	Much more	1 ○	2 ○	3 ○	4 ○	5 ○	6 ○	7 ○	8 ○	9 ○	Much less
16.	The patient showed ….. fluid movement than before deep brain stimulation started	**Much less**	1 ○	2 ○	3 ○	4 ○	5 ○	6 ○	7 ○	8 ○	9 ○	**Much more**
17.	The patient showed ….. angry outbursts than before deep brain stimulation started	Many more	1 ○	2 ○	3 ○	4 ○	5 ○	6 ○	7 ○	8 ○	9 ○	Many fewer
18.	The patient showed ….. depression than before deep brain stimulation started	Much more	1 ○	2 ○	3 ○	4 ○	5 ○	6 ○	7 ○	8 ○	9 ○	Much less
19.	The patient showed ….. change in personality compared to before deep brain stimulation started	A major	1 ○	2 ○	3 ○	4 ○	5 ○	6 ○	7 ○	8 ○	9 ○	No
20.	According to *the patient's opinion,* deep brain stimulation for Lesch-Nyhan disease is ……	Very effective	1 ○	2 ○	3 ○	4 ○	5 ○	6 ○	7 ○	8 ○	9 ○	Use-less
